# Nutrition Education, Understanding, and Counseling Practices Among Physiatrists: A Survey Study

**DOI:** 10.7759/cureus.28756

**Published:** 2022-09-03

**Authors:** Jessica M Calandra, Frances S Shofer, Ariana M Chao, Randel L Swanson

**Affiliations:** 1 Physical Medicine and Rehabilitation, MossRehab, Albert Einstein Healthcare Network, Elkins Park, USA; 2 Physical Medicine and Rehabilitation, University of Pennsylvania Perelman School of Medicine, Philadelphia, USA; 3 Emergency Medicine, University of Pennsylvania Perelman School of Medicine, Philadelphia, USA; 4 Biobehavioral Health Sciences, University of Pennsylvania School of Nursing, Philadelphia, USA; 5 Psychiatry, University of Pennsylvania Perelman School of Medicine, Philadelphia, USA; 6 Center for Neurotrauma, Neurodegeneration and Restoration, Cpl. Michael J. Crescenz VA Medical Center, Philadelphia, USA

**Keywords:** public health, physical and rehabilitation medicine, continuing education, medical education, nutrition

## Abstract

Introduction: Nutritional counseling is relevant to physiatry practice. However, physiatrists’ nutrition knowledge base and their frequency of incorporating nutritional education into routine clinical encounters are currently unknown. The objective of this study was to assess physiatrists’ nutrition education, nutrition knowledge, willingness to implement nutrition counseling in clinical practice, and perceived barriers to providing nutritional counseling to patients, using an online survey. The hypothesis was that few physiatrists offer dietary counseling to their patients on a routine basis and that barriers likely include time constraints in a typical office visit, lack of provider confidence in providing dietary recommendations, and lack of provider reimbursement.

Methods: This study was a cross-sectional online survey that was distributed via email to a convenience sample of 179 resident, fellow, and attending physiatrists associated with two major academic institutions. The survey consisted of 26 questions regarding demographics, nutrition counseling practices and attitudes, basic nutrition knowledge, and perceived barriers to providing nutrition counseling.

Results: Of 59 participants, 78% reported receiving education in nutrition and/or behavioral counseling in medical school. In contrast, 39% of participants did not feel adequately trained to discuss nutrition and diet-related issues with patients. Barriers to providing nutritional counseling were time constraints (83%), socioeconomic factors outside of patients’ control preventing them from adhering to a healthier diet (76%), and not having enough nutrition knowledge to do so appropriately (62%). Respondents (86%) either agreed or strongly agreed that additional training in nutrition would allow them to provide better clinical care in the prevention of progressive cardiovascular and neurovascular disease. Further, respondents (85%) either agreed or strongly agreed that they would be interested in a web-based continuing medical education training in nutrition behavioral counseling and evidence-based dietary recommendations.

Conclusions: The findings refuted the hypothesis that few physiatrists in the sample offer dietary counseling to their patients on a routine basis. Results demonstrated that many of them acknowledge its relevance and would be interested in further education on the subject. Physiatrists also appear to have perceived barriers to offering nutritional counseling, but some of them varied by the amount of medical experience. Based on these findings, this study demonstrated that it would be worthwhile to develop such a continuing medical education activity with a focus on populations commonly treated by physiatrists.

## Introduction

The prevalence of conditions that increase cardiovascular risk such as type 2 diabetes mellitus and overweight/obesity has been increasing [1-5]. The prevalence of diabetes mellitus among US adults has increased from 9.8% in 1999-2000 to 14.3% in 2017-2018 [2-4]. Further, the prevalence of obesity in US adults has increased from 30.5% in 1999-2000 to 42.4% in 2017-2018 [5]. Clinical guidelines [6-8] for cardiovascular disease, type 2 diabetes, and obesity highlight the importance of modifiable lifestyle behaviors, such as diet, physical activity, smoking, and sleep in the prevention and treatment of these conditions. Further, lifestyle modifications help improve physical [9-11] and cognitive function [12,13]. Unfortunately, practicing providers today likely receive little to no formal nutrition education. For example, the US National Research Council Committee on Nutrition Education recommended a minimum of 25-30 nutrition classroom hours in 1985 [14], but a survey of 121 US medical schools revealed a mean of 19.0 hours dedicated to this during the 2012-2013 academic year, with only 29% of the participating medical schools meeting the minimum [15]. Spencer and colleagues surveyed 2316 US medical students at three different times during their schooling and found that during their final year, 46% felt nutrition counseling was relevant to their intended practice, down from 72% during their first year. Spencer’s group concluded that intention to practice primary care was predictive of students finding nutrition counseling relevant and practicing it more frequently [16].

While studies have generally examined nutrition education among physicians, nurse practitioners, and other healthcare professionals [17], little is known about the nutrition education physiatrists receive. Physiatrists care for patients with functional impairments from various disabling conditions. Nutrition plays an important role in promoting functional independence, such as in preventing and treating obesity [18-20], but it is unclear how prepared physiatrists feel to provide nutrition counseling to patients. Polak and colleagues [21] note that nutrition is rarely considered in the specialty’s literature, and introduce basic concepts in nutrition and behavioral counseling to physiatrists, emphasizing that they need additional education on the subject in order to gain expertise.

The purpose of this study was to assess physiatrists’ prior nutrition education, nutrition knowledge, willingness to implement nutrition counseling in clinical practice, and perceived barriers to providing nutritional counseling to patients. Based on previous research on other healthcare providers [22,23], it was hypothesized that few physiatrists offer dietary counseling to their patients on a routine basis and that barriers likely include time constraints in a typical office visit, lack of provider confidence in providing dietary recommendations, and lack of provider reimbursement. A secondary objective was to examine duration in physiatric practice as a potential factor related to attitudes regarding nutrition counseling.

The preliminary results of this survey study were previously presented as a meeting abstract and poster at the 2022Association of Academic Physiatrists(AAP) Annual Meeting (*Physiatry '22*) on May 26, 2022. The abstract was subsequently published in the American Journal of Physical Medicine & Rehabilitation's July supplement containing the "*Abstracts of Scientific Papers and Posters Presented at Physiatry ’22*" [Calandra JM, et al.: Am J Phys Med Rehabil 101(7 (Suppl)): a88, July 2022].

## Materials and methods

Study design

A cross-sectional online survey was distributed via email between May 10th and June 14th, 2021, to the resident, fellow, and attending physiatrists associated with two major academic institutions with Physical Medicine and Rehabilitation (PM&R) residency programs in Philadelphia, PA.

Study population

The study population consisted of a convenience sample of resident, fellow, or attending physicians in PM&R. Those not currently practicing and prospective physiatrists in internship were excluded. Participants self-selected by responding to an email to complete the survey. This study was deemed exempt by the University of Pennsylvania Institutional Review Board. 

Survey tool

The survey consisted of 26 questions regarding demographics, nutrition counseling practices and attitudes, basic nutrition knowledge, and perceived barriers to providing nutrition counseling (see Appendixfor the administered survey). The estimated time to complete the questionnaire was no more than 15 minutes. The questionnaire was adapted from a previous survey study by Harkin et al. [22]. The survey was pilot tested prior to implementation. After a written explanation of the purpose, benefits, and risks of the survey, participants consented to participation electronically.

Survey measures

The measures included the following four domains: demographics and clinical practice, nutrition counseling practices and attitudes, nutrition knowledge, and barriers to nutrition counseling. 

Demographics and Clinical Practice

Demographic and clinical practice variables including age, gender identity, stage of career, years in practice since training, practice environment, and foci of physiatry practice were collected.

Nutrition Counseling Practices and Attitudes

In this domain, participants answered items describing settings in which they might have received prior nutrition education, incorporation of nutrition counseling in practice, attitudes about the relevance of nutrition to their practice, perceptions about their abilities to counsel patients on nutrition, perceptions about their effectiveness in helping patients improve their diet, perceived importance of various modifiable lifestyle factors, the proportion of time spent during a routine encounter providing diet and/or lifestyle counseling, the proportion of patients they refer to allied professionals to implement these changes, and attitudes about further nutrition education. One item queried diets routinely recommended to patients, and if a diet was not identified in the choices, participants could specify it in a subsequent open-ended item. The respondents' perceived importance of six separate modifiable lifestyle factors were answered in a 1-10 point Likert scale format within one item.

Nutrition Knowledge 

This domain featured two multiple-choice items and one true/false item testing basic dietary knowledge.

Barriers to Nutrition Counseling

Participants were presented with statements about potential barriers to providing nutrition counseling and were asked to respond using a Likert scale from 1 to 5 (1 = strongly agreed, 3 = neutral, 5 = strongly disagreed) toward these statements.

Statistical analysis

Standard summary statistics such as frequencies and percentages, means with standard deviations (SD), or medians with interquartile range (IQR) were used to describe the data. To determine differences in the perceived importance of modifiable lifestyle factors and barriers to nutrition counseling, by years of practice (in training, 1-15 years, >15 years), a one-way analysis of variance was performed. For barriers to counseling, the Likert scales were flipped for ease of comprehension, so that strongly disagree = 1 and strongly agree = 5. To adjust for multiple post-hoc pairwise comparisons, Tukey-Kramer tests were used. All analyses were performed using SAS statistical software (Version 9.4, SAS Institute, Cary NC).

## Results

Participant demographics

Of the 179 physiatrists invited to participate in the study, 59 (33.0%) respondents completed all survey items. The median participant age was 35 years (IQR: 31-46.5) and 35 (59.3%) of the participants identified as male. Of the respondents, 52.5% were attending physicians, 37.3% were residents, and 10.2% were fellows. For practice experience, 28 (47.5%) respondents were still in training, 3 (5.1%) had fewer than five years, and 28 (14 in each, 23.7%) 5-15 years, or greater than 15 years of experience. For the primary practice environment, 78.0% of respondents were within an academic medical center, 15.3% were in private practice, and 6.8% were in community hospital-based practice. The most common practice focus among respondents was general physiatry (62.7%), followed by brain injury medicine or stroke rehabilitation (32.2% each), interventional pain/spine rehabilitation (30.5%), spasticity management, and pain medicine or gait evaluation (27.1% each, Table 1).

**Table 1:** Respondents’ Foci of Physiatry Practice Note: respondents could report more than one focus of clinical practice.

Focus of Physiatry Practice	No. of Respondents (%)
General	37 (62.7)
Brain Injury Medicine	19 (32.2)
Stroke Rehabilitation	19 (32.2)
Interventional Pain/Spine Rehabilitation	18 (30.5)
Spasticity Management	16 (27.1)
Gait Evaluation/Orthotics	16 (27.1)
Pain Medicine	16 (27.1)
Electrodiagnostics	15 (25.4)
Spinal Cord Injury Medicine	14 (23.7)
Sports Medicine	14 (23.7)
Amputee Rehabilitation/Prosthetics	11 (18.6)
Cardiac Rehabilitation	8 (13.6)
Pediatric Rehabilitation	7 (11.9)
Palliative Care	3 (5.08)

Nutrition counseling practices and attitudes

Most respondents (78.0%) reported having received education in nutrition and/or behavioral counseling in medical school; 3.4% reported not having had any of this education. While 84.7% of respondents felt discussing nutrition and diet-related issues with patients was relevant to their practice and 81.4% felt that they could be at least slightly effective in helping patients improve their diet, 39.0% of participants did not feel adequately trained to discuss nutrition and diet-related issues with patients. In response to the diet(s) recommended, the most common response was that they do not routinely recommend any specific diet(s) (42.4%). Of those who recommended diets, the Mediterranean diet (28.8%) was the most common, followed by a low-carbohydrate diet such as the Atkin's diet (16.9%), and intermittent fasting (16.9%, Table 2).

**Table 2:** Diets Routinely Recommended by Respondents *Specified by one respondent as gluten-free and dairy-free.

Diets Recommended to Patients	No. of Respondents (%)
Mediterranean	17 (28.8)
Low-Carbohydrate (e.g. Atkin’s)	10 (16.9)
Intermittent Fasting	10 (16.9)
Low-Fat	8 (13.6)
Vegetarian	8 (13.6)
Low Glycemic Index (e.g. South Beach, Zone)	6 (10.2)
Dietary Approaches to Stop Hypertension (DASH)	4 (6.8)
Mediterranean-DASH Intervention for Neurodegenerative Delay (MIND)	4 (6.8)
Vegan	2 (3.4)
Portion Control	2 (3.4)
WW (Weight Watchers)	2 (3.4)
Reduced Caloric Intake With Increased Exercise	2 (3.4)
Anti-inflammatory Diet*	2 (3.4)
Reducing Refined Sugars	1 (1.7)
Very Low-Fat (e.g. Ornish)	1 (1.7)
High Protein	1 (1.7)
Non-processed Foods	1 (1.7)
Patient-Specific	1 (1.7)
Rare Adipose Disorder (RAD) Diet	1 (1.7)
Addition of Vegetables	1 (1.7)

Regarding dietary and lifestyle counseling practices, most respondents (72.9%) reported that they spend approximately 1-10% of their visits counseling patients about diet and/or lifestyle interventions to prevent cardiovascular and neurovascular disease. None of the respondents reported spending more than 30% of their encounters on this.

**Figure 1:**
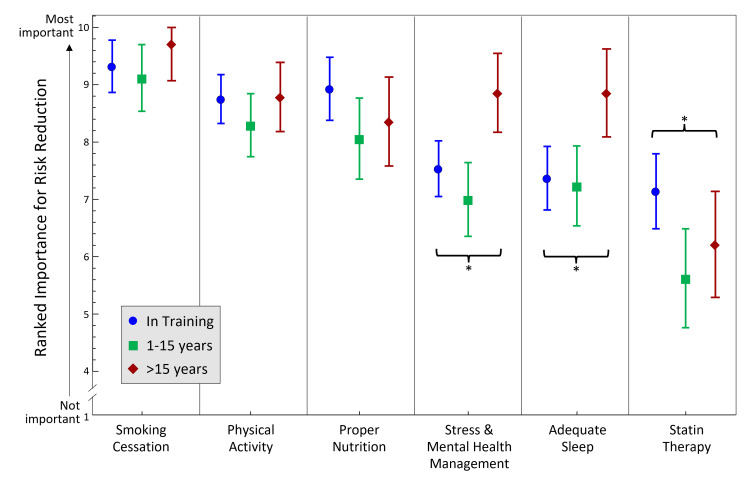
Respondents' Perceived Importance of Six Separate Modifiable Lifestyle Factors Each sub-question was answered using a 1-10 point Likert scale format within one survey item. * = p < 0.05; "In training (blue)" = physiatry residents or physiatrists in sub-specialty fellowship; "1-15 years (green)" = attending physiatrists in practice between 1-15 years; ">15 years (red)" = attending physiatrists in practice for more than 15 years.

Among the modifiable lifestyle factors, the respondents ranked smoking cessation the highest importance based on a mean of 9.4 ± 1.2, followed by physical activity (8.6 ± 1.1), proper nutrition (8.5 ± 1.5), stress/mental health management (7.7 ± 1.4), adequate sleep (7.7 ± 1.6), and statin therapy (6.50 ± 1.8). Although statin therapy was ranked the least important, trainees were more likely to rank statin therapy higher in importance (mean of 7.1) compared to those practicing independently (mean of 6.2 for those in practice >15 years and 5.6 for those in practice 1-15 years, p = 0.02, Figure 1). In contrast, those with >15 years of experience were more likely to rank adequate sleep higher than the less experienced groups (mean of 8.9 vs 7.2 for those in practice 1-15 years and 7.4 for those still in training, p = 0.004). Finally, respondents with >15 years of experience were more likely to rank stress/mental health management higher than those with <15 years of experience (mean of 8.9 vs 7.0 for those 1-15 years in practice and 7.5 for those still in training, p = 0.0007). There were no significant differences in the perceived importance of physical activity, smoking cessation, and proper nutrition among the respondents grouped by time practicing physiatry.

Importantly, 86.4% of respondents either agreed or strongly agreed that additional training in nutrition would allow them to provide better clinical care in the prevention of cardiovascular and neurovascular disease. Similarly, 84.7% of respondents either agreed or strongly agreed that they would be interested in a web-based continuing medical education (CME) training in nutrition behavioral counseling and evidence-based dietary recommendations.

Basic nutrition knowledge

Of respondents, 84.7% knew that the Mediterranean diet was shown to reduce cardiovascular events in a randomized controlled trial [24]. Similarly, 70.7% knew that the Mediterranean-DASH Intervention for Neurodegenerative Delay (MIND) diet was associated with a reduced incidence of Alzheimer’s disease in a prospective longitudinal cohort study [25]. Almost all respondents (98.3%) knew of the relationship between cardiovascular and neurovascular health, as queried in item 21 of the survey (see Appendixfor the administered survey).

Barriers to nutrition counseling

**Figure 2:**
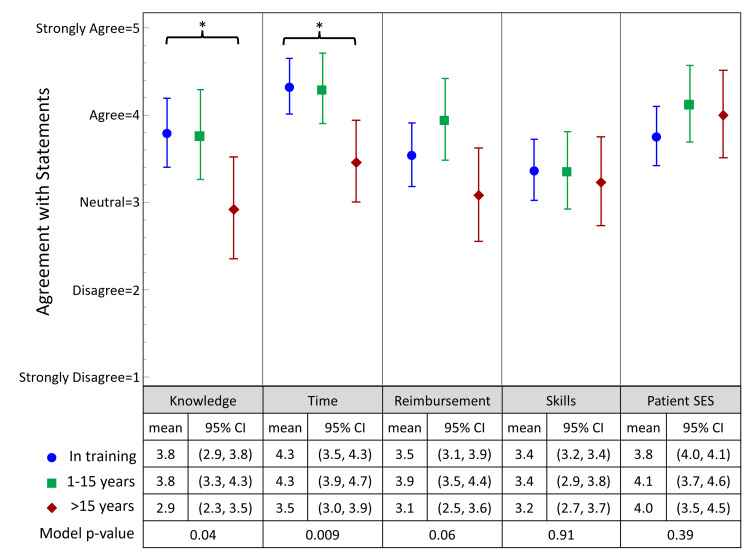
Barriers to Nutrition Counseling Participants were presented with statements about potential barriers to providing nutrition counseling and were asked to respond using a Likert scale from 1 to 5 toward these statements. To determine differences in perceived importance of modifiable lifestyle factors and barriers to nutrition counseling, by years of practice (in training [resident or fellow], 1-15 years, >15 years), one-way analysis of variance was performed.  As detailed in the Methods section, the Likert scales were flipped during data analysis compared to that on the survey for ease of comprehension, so that strongly disagree = 1 and strongly agree = 5. * = p < 0.05; "In training (blue)" = physiatry residents or physiatrists in sub-specialty fellowship; "1-15 years (green) = attending physiatrists in practice between 1 and 15 years; ">15 years (red)" = attending physiatrists in practice for more than 15 years. CI, confidence interval; SES, socioeconomic status.

All groups felt insufficient behavioral counseling skills, inadequate potential reimbursement, or socioeconomic factors outside of patients’ control were barriers to providing nutritional counseling. Those in practice longer were more likely to disagree with time, reimbursement, or knowledge being a barrier (Figure 2). Those practicing >15 years were more likely to disagree with time constraints being a barrier to providing nutritional counseling, in contrast to those practicing <15 years (mean of 2.5 vs 1.7 for those in practice 1-15 years and still in training, p = 0.009). Those practicing >15 years were also more likely to disagree with insufficient nutrition knowledge as a barrier (mean 3.1 vs 2.2 for those in practice 1-15 years and still in training, p = 0.0427). 

## Discussion

The purpose of this cross-sectional survey study was to characterize nutrition education, practices, attitudes, and perceived barriers to nutritional behavioral counseling among physiatrists. In contrast to the hypothesis, it was found that 93.2% of the physiatrists spend at least 1% of their time during a routine encounter counseling patients on diet and/or lifestyle changes. When asked to estimate the proportion of time spent doing this, however, the majority (72.9%) selected 1-10% of the encounter, while none selected 31-40% or greater. This is not entirely surprising, as patients receiving physiatric care have numerous complex needs, such as evaluation of adaptive equipment or gait, which require significant attention.

Indeed, when given the opportunity to select diets routinely recommended in their clinical practice, 42.4% of respondents reported that they do not routinely recommend any specific diet(s). This result contrasts with a similar survey item administered by Harkin et al. to cardiologists and internists - only 16% of their respondents were in this category [22]. These findings highlight a potentially significant difference in the practice pattern of physiatrists compared to cardiologists and internists.

Interestingly, none of the respondents disagreed with the statement “I feel that discussing nutrition and diet-related issues with patients is relevant to my practice,” but in response to whether respondents felt adequately trained to discuss these issues with patients, responses were relatively discordant, mostly distributed among selections of “agree,” “neutral,” and “disagree.” Even though only 35.6% agreed or strongly agreed with this statement, this is still more than double the number of internists and cardiologists who responded this way to the analogous item in Harkin et al., which was 13.5% [22].

In terms of perceived effectiveness in helping patients improve diet, almost half of the respondents (49.2%) felt they could be slightly effective, with the other half of responses among “very effective,” “effective,” and “not very effective.” This may indicate that there is a mismatch between physiatrists’ perception of nutrition relevance to their practice and their perception of their ability to use it to help patients make lifestyle improvements. This study demonstrates that a considerable number of physiatrists (39.0%) do not feel adequately trained to discuss nutrition and diet-related issues with patients, similar to many cardiologists and internists as shown by Harkin’s survey [22].

It should be noted that an overwhelming majority (86.5%) of the respondents either agreed or strongly agreed with the statement “additional training in nutrition would allow me to provide better clinical care in the prevention of cardiovascular/neurovascular disease.” Similarly, 84.8% agreed or strongly agreed with the statement “I would be interested in a web-based CME training in nutrition behavioral counseling and evidence-based dietary recommendations.”

The respondents’ performance on the nutrition knowledge questions shows that most of the respondents already have at least some basic familiarity with the subject, as >70% of the respondents answered 3/3 questions correctly. These questions may serve as a starting point in the development of potential nutrition CME with a physiatrist target audience. Physiatrists would greatly benefit from CME specifically designed for their specialty due to the diverse and medically complex populations they serve. 

Regarding ranking the importance of the modifiable lifestyle factors, the highest three ranked lifestyle factors (smoking cessation, physical activity, and proper nutrition) may be reflective of the ones most often addressed by this group. Indeed, these factors did not significantly differ in rank based on respondents’ time practicing physiatry. 

A secondary objective of this study was to assess duration in physiatric practice as a factor in lifestyle counseling. Several differences were found in the perceived importance of various modifiable lifestyle factors by duration in practice. The more seasoned physiatrists tended to rank stress/mental health management and adequate sleep higher than those with <15 years in practice. It is unclear why more experienced physicians recognize these factors as more important than their less experienced counterparts; perhaps it reflects their experience with patients, themselves, or both. 

The in-training physiatrists tended to rank statin therapy as more important than those with more experience did. A potential explanation could be trainees’ knowledge of recent evidence of statin use’s association with decreased risk of all-cause and cardiovascular mortality in a large retrospective cohort study in US veterans 75 years and older [26]. Perhaps more experienced physiatrists are considering modifiable lifestyle factors’ importance in the context of a patient's quality of life rather than potential associations with mortality.

The most common barriers to offering dietary counseling in this study include time constraints in a typical office visit (endorsed by 84.5% of the sample), socioeconomic factors outside of patients’ control preventing them from adhering to a healthier diet (endorsed by 77.5% of the sample), and lack of provider confidence in providing dietary recommendations (endorsed by 62.1% of the sample). 

Some differences in perceived barriers to practicing nutritional behavioral counseling were identified by the duration of physiatric practice. The physiatrists in practice with <15 years were more likely to agree with time constraints as a barrier to providing nutritional counseling to patients, while those with >15 years’ experience tended to disagree with time constraints as a barrier. A survey administered to primary care providers by Kushner revealed that lack of time was the top perceived barrier by its respondents, perhaps because there are often a variety of topics to cover in this setting, such as tracking age-appropriate screenings and vaccinations, in addition to a patient’s individual medical problems [23]. The physiatrists with >15 years of experience tended to disagree with insufficient nutrition knowledge as a barrier, potentially reflecting their confidence in counseling over time. Among all experience groups, respondents tended to agree that socioeconomic factors beyond patients’ control were a barrier to providing nutritional counseling. A CME event targeted at physiatrists could also be used to troubleshoot ways to break through the barriers of putting nutrition knowledge into practice in diverse socioeconomic environments.

Study limitations

This study had a number of limitations. First, the study population was a convenience sample, as the prospective participants were associated with either of two PM&R training programs in the same city. Further, given that the participants were associated with urban PM&R training programs in one city, the study population is likely not reflective of the physiatrist population in the US. Finally, the sample size is small. There were 66 individuals who gave consent and proceeded with the questionnaire, but 59 completed it in full. Although this may appear as a study weakness, the authors felt that allowing some questionnaire items to be optional would reduce respondent fatigue and maximize participation.

## Conclusions

This study refuted the hypothesis that few physiatrists in the sample offer dietary counseling to their patients on a routine basis. It was discovered that many of them acknowledge its relevance in their specialty and would be interested in further education on the subject. Physiatrists also appear to have perceived barriers to offering nutritional counseling, but some of them varied by the amount of medical experience, including time constraints, lack of nutrition knowledge, and socioeconomic factors outside of patients’ control. Future research should include developing a CME program on nutrition and its related behavioral counseling, with physiatrists both in training and practice as the target audience, followed by an analysis of the educational activity for effectiveness in practice. 

## Appendices

Supplemental material - administered survey

Nutrition Education, Understanding, and Counseling Practices Among Physiatrists

SCREENING QUESTION GIVEN PRIOR TO SURVEY CONSENT FORM (positive response to “intern” or “retiree” is equivalent to meeting exclusion criteria and these individuals will be thanked for their time and notified that they meet exclusion criteria and need not proceed with the survey):

*Please select the current stage of your career:  Intern, Resident, Fellow, Attending, Retiree

Demographics and Professional Information:

1) Please enter your age in years: _____

2) Please select your gender: *Male; Female; Transgender; Gender nonbinary; Intersex; Prefer not to say*

3)* Please select the current stage of your career: *Resident; Fellow; Attending*

4) What areas make up the focus of your physiatry practice? Select all that apply: *General Physical Medicine and Rehabilitation; Brain Injury Medicine; Stroke Rehabilitation; Spinal Cord Injury Medicine; Sports Medicine; Electrodiagnostics; Amputee Rehabilitation/Prosthetics; Spasticity Management; Gait Evaluation/Orthotics; Palliative Care; Pediatric Rehabilitation; Interventional Pain/Spine Rehabilitation; Pain Medicine; Cardiac Rehabilitation; Other*

5) If you selected “other,” please define the focus of your physiatry practice: _____

6)* How many years have you been in medical practice since completing training? *I am still in training; <5 years; 5-15 years, >15 years*

7)* Select your primary practice environment: *Private practice; Academic medical center-based practice; Community hospital-based practice*

Dietar**y** Counseling Practices and Attitudes:

8) In what settings have you received education in nutrition and/or behavioral counseling? Select all that apply: *In undergraduate or graduate course(s) prior to medical school; In class(es) during medical school; In clinical rotation(s) during medical school; In residency during didactics; In residency during clinical practice; In continuing medical education course(s); In graduate or certificate course(s) completed during training or practice; In self-directed learning, i.e. independent review of literature; In my research activities; I have not had any form of this education*

9)* Which diet(s) do you routinely recommend in your clinical practice? Select all that apply: *Low-carbohydrate (e.g. Atkin's); Low-fat; Very low fat (Ornish); Low glycemic index (e.g. South Beach, Zone); Mediterranean; Dietary Approaches to Stop Hypertension (DASH); Mediterranean-DASH Intervention for Neurodegenerative Delay (MIND); Intermittent fasting; Vegetarian; Vegan; Other; I don't recommend specific diets in my practice*

10) If you selected “other,” please specify which other diet(s) you routinely recommend in your clinical practice: _____

11)* I feel that I am adequately trained to discuss nutrition and diet-related issues with patients: *Strongly agree; Agree; Neutral; Disagree; Strongly disagree*

12) I feel that discussing nutrition and diet-related issues with patients is relevant to my practice: *Strongly agree; Agree; Neutral; Disagree; Strongly disagree*

13)* How effective do you feel you can be in helping patients to improve their diet? *Very effective; Effective; Slightly effective; Not very effective*

14)* Approximately what proportion of your time during a routine encounter do you personally spend counseling your patients about diet and/or lifestyle changes to prevent cardiovascular and neurovascular disease, if applicable? *None; 1-10%; 11-20%; 21-30%; 31-40%; 41-50%; More than 50%*

15)* Of your patients who have suboptimal nutritional habits, what percentage do you refer to a nutritionist, registered dietitian, or a weight loss program? *<1%; 1-10%; 11-20%; 21-30%; >30%; I don't refer to a nutritionist, registered dietitian or weight loss program; My patients do not have suboptimal nutritional habits*

16)* On a scale of 1-10, with 10 being the most important, how important do you believe each of the following factors are for cardiovascular/neurovascular risk reduction: *Proper nutrition (1 2 3 4 5 6 7 8 9 10); Physical activity (1 2 3 4 5 6 7 8 9 10); Statin therapy (1 2 3 4 5 6 7 8 9 10); Smoking cessation (1 2 3 4 5 6 7 8 9 10); Adequate sleep (1 2 3 4 5 6 7 8 9 10); Stress/mental health management (1 2 3 4 5 6 7 8 9 10)*

17)* Additional training in nutrition would allow me to provide better clinical care in the prevention of cardiovascular/neurovascular disease.* Strongly agree; Agree; Neutral; Disagree; Strongly disagree*

18) I would be interested in a web-based CME training in nutrition behavioral counseling and evidence-based dietary recommendations. *Strongly agree; Agree; Neutral; Disagree; Strongly disagree*

Basic Nutrition Knowledge:

19)* Which of the following diets has been demonstrated to reduce cardiovascular events in randomized controlled trial(s)? *Low-carbohydrate (i.e. Atkin's); Low-fat; Low glycemic index (i.e. South Beach, Zone); Mediterranean; Vegan*

20)* Which of the following diets was found to be associated with reduced incidence of Alzheimer's disease in a prospective longitudinal cohort study? *Low glycemic index (i.e. South Beach, Zone); Mediterranean; Dietary Approaches to Stop Hypertension (DASH); Mediterranean-DASH Intervention for Neurodegenerative Delay (MIND)*

21) True or false: There is a relationship between cardiovascular and neurovascular health. *True; False*

Barriers to Nutrition Counseling **:**

22) I would be interested in talking to my patients more about implementing a healthy diet, but I feel there is not enough time to do so in a routine visit. *Strongly agree; Agree; Neutral; Disagree; Strongly disagree*

23) I would be interested in talking to my patients more about implementing a healthy diet, but I feel I do not have enough nutrition knowledge to do so appropriately.* Strongly agree; Agree; Neutral; Disagree; Strongly disagree*

24) I would be interested in talking to my patients more about implementing a healthy diet, but I feel I do not have enough behavioral counseling skills to do so appropriately. *Strongly agree; Agree; Neutral; Disagree; Strongly disagree*

25) I would be interested in talking to my patients more about implementing a healthy diet, but I feel there is inadequate potential reimbursement for this clinical task. *Strongly agree; Agree; Neutral; Disagree; Strongly disagree*

26) I would be interested in talking to my patients more about implementing a healthy diet, but I feel that there are socioeconomic factors outside of my patients' control preventing them from adhering to a healthier diet. *Strongly agree; Agree; Neutral; Disagree; Strongly disagree*

Notes

*Questions either taken directly or adapted from Harkin et al. [22].

Survey modified from Harkin et al. [22]. Copyright © 2018 by the authors.  Reprinted with permission of SAGE Publications.
